# Using NGS Technology and Association Mapping to Identify Candidate Genes Associated with Fusarium Stalk Rot Resistance

**DOI:** 10.3390/genes15010106

**Published:** 2024-01-16

**Authors:** Jan Bocianowski

**Affiliations:** Department of Mathematical and Statistical Methods, Poznań University of Life Sciences, Wojska Polskiego 28, 60-637 Poznań, Poland; jan.bocianowski@up.poznan.pl; Tel.: +48-61-8487143

**Keywords:** maize, next-generation sequencing, SNP markers, SilicoDArT markers, stalk rot

## Abstract

Stalk rot caused by *Fusarium* fungi is one of the most widespread and devastating diseases of maize, and the introduction of resistant genotypes is one of the most effective strategies for controlling the disease. Breeding genotypes with genetically determined resistance will also allow less use of crop protection products. The aim of the research was to identify molecular markers and associated candidate genes determining maize plant resistance to Fusarium stalk rot. The plant material for this study consisted of 122 maize hybrids. The experiment was conducted in two localities: Smolice and Kobierzyce. The Fusarium stalk rot values ranged from 1.65% (for genotype G01.10) to 31.18% (for genotype G03.07) in Kobierzyce and from 0.00% (for 58 genotypes) to 6.36% (G05.03) in Smolice. The analyzed genotypes were simultaneously subjected to next-generation sequencing using the Illumina platform. Illumina sequencing identified 60,436 SilicoDArT markers and 32,178 SNP markers (92,614 in total). For association mapping, 32,900 markers (26,234 SilicoDArT and 6666 SNP) meeting the criteria (MAF > 0.25 and the number of missing observations <10%) were used. The results of the observation of the degree of infection and sequencing were used for association mapping, which ultimately resulted in the selection of ten molecular markers important at both places. Among the identified markers, two SNP markers that are located inside candidate genes play an important role. Marker 4772836 is located inside the serine/threonine-protein kinase bsk3 gene, while marker 4765764 is located inside the histidine kinase 1 gene. Both genes can be associated with plant resistance to Fusarium stalk rot, and these genes can also be used in breeding programs to select resistant varieties.

## 1. Introduction

In the last decade, the increase in temperature, expansion of areas, intensification of cultivation, introduction of agrotechnical simplifications, and the appearance of new pest species have resulted in a significant increase in the threat to the amount and quality of maize yields [1]. Crops of this cereal are frequently infected by pathogenic fungi, resulting in serious yield losses [2]. Currently, fungi of the genus *Fusarium* can be classified as the most dangerous pathogens causing serious losses in maize plantations. The fungus *Fusarium graminearum* causes stalk rot in maize [3,4]. This pathogen not only causes yield losses, but also poses a huge threat related to the presence of mycotoxins [5,6,7]. Fungi of the genus *Fusarium* colonize soil and crop residues of maize and cereals, and are also present on the surface of grains. They reach plants directly from resting places and through wind, water, soil fragments (dust), insects, and animals. Maize is exposed to *Fusarium* fungi from the moment the grain is sown [8]. As a result of infection, maize plants often lodge, leading to a decline in planting density.

Maize plant lodging caused by *Fusarium* fungus infection is often observed in fields where seed is sown untreated with a fungicide. This causes large losses in the quantity and quality of the crop. For some genotypes, these losses are even 100 percent. The development of the pathogen can also be favored by weather factors, such as a cool and rainy spring, heavy rainfall, or sowing too deeply in heavy soil [9]. Infection can be prevented; several studies have shown that soil fertilization has a positive effect on plant tolerance to pathogenic fungi, particularly the use of fertilizers containing zinc or potassium phosphite [10,11,12]. Stalk rot usually occurs when plant tissues are damaged by *Ostrinia nubilalis*. The feeding of this pest is largely responsible for the development of the disease, especially since *O. nubilalis* is also a vector of this fungus and, migrating between plants, transfers fungal spores from diseased plants to healthy ones [13]. There are usually two generations of *O. nubilalis* larvae per year: the first generation attacks plants in the middle or end of the vegetative phase, and the second generation attacks plants in the reproductive phase (from early milk stage to maturity) [14]. Strong decomposition of stem tissues, including nodes, leads to whole plants falling over, making harvesting difficult and sometimes impossible.

Fighting *Fusarium* diseases that appear at the end of the growing season is difficult, especially when no treatments are applied beforehand to directly or indirectly limit infection [15]. Currently, the most effective method of combating this pathogen is the cultivation of varieties with genetically determined resistance to *F. graminearum*. A dynamically changing climate and an increase in demand for maize, driven by a natural increase in population, are catalysts for research into genes and genomic regions important to agronomy [16,17]. Scientists have long used next-generation sequencing (NGS) and association mapping methods to identify markers associated with resistance genes. Genome-wide association studies (GWAS) are a useful tool for identifying candidate genes, especially when combined with QTL mapping to validate loci for quantitative traits. Zila et al. [18,19] performed GWAS tests in maize to detect SNPs associated with increased resistance to *Fusarium*. They identified 10 SNP markers significantly associated with resistance to this pathogen. Many previous studies have been conducted with GWAS and SNPs on Fusarium stalk rot. Liu et at. [20], based on 165 maize inbred lines and 4666 SNP markers, performed genetic diversity analysis, GWAS, linkage analysis, and phenotypic analysis through the inoculation of stalk rot pathogen on maize plants in the field. For identification and validation of genomic regions associated with Fusarium stalk rot resistance, Rashid et al. [21] used GWAS for 342 maize lines and 290,626 SNPs. The panel was screened for Fusarium stalk rot in three environments using standard artificial inoculation methodology. In GWAS conducted by Stagnati et al. [22] on 230 inbred lines, some of the SNPs identified for seedling rot co-localized within QTL intervals previously identified for Fusarium seed rot, Fusarium ear rot, and fumonisin accumulation in maize. The introduction of molecular analyses made it possible to develop a methodology for genetic marker-assisted selection (MAS). The scope of application of this methodology depends on the progress of knowledge of the genome of the species [23,24]. The completion of the sequencing of the human genome has opened up a wide range of possibilities for understanding the genomic sequences of crop plants [25,26,27,28]. For several years, maize breeding around the world has been supported by useful molecular markers. Many authors state in their publications that breeding supported by molecular marker accelerates yield increases not only in the United States, but also in other countries, offering great potential to increase the productivity and value of maize germplasm [29,30].

Therefore, the aim of this study was to identify new SNP and SilicoDArT markers linked to candidate genes for maize stalk rot resistance.

## 2. Materials and Methods

### 2.1. Plant Material

The plant material used in this study came from Smolice Plant Breeding Ltd. (Smolice, Poland) IHAR Group (51°42′12″ N 17°10′10″ E) and Małopolska Plant Breeding Ltd. (Kobierzyce, Poland) (50°58′17″ N 16°55′50″ E). The plant material included 122 maize hybrids (G01.01, G01.02, G01.03, G01.04, G01.05, G01.06, G01.07, G01.08, G01.09, G01.10, G01.11, G01.12, G01.13, G01.14, G01.15, G01.16, G01.17, G01.18, G01.19, G01.20, G01.21, G02.01, G02.02, G02.03, G02.04, G02.05, G02.06, G02.07, G02.08, G02.09, G02.10, G02.11, G02.12, G02.13, G02.14, G02.15, G02.16, G02.17, G02.18, G02.19, G02.20, G02.21, G03.01, G03.02, G03.03, G03.04, G03.05, G03.06, G03.07, G03.08, G03.09, G03.10, G03.11, G03.12, G03.13, G03.14, G03.15, G03.16, G03.17, G03.18, G03.19, G03.20, G03.21, G04.01, G04.02, G04.03, G04.04, G04.05, G04.06, G04.07, G04.08, G04.09, G04.10, G04.11, G04.12, G04.13, G04.14, G04.15, G04.16, G04.17, G04.18, G04.19, G04.20, G04.21, G05.01, G05.02, G05.03, G05.04, G05.05, G05.06, G05.07, G05.08, G05.09, G05.10, G05.11, G05.12, G05.13, G05.14, G05.15, G05.16, G05.17, G05.18, G05.19, G05.20, G05.21, G06.01, G06.02, G06.03, G06.04, G06.05, G06.06, G06.07, G06.08, G06.09, G06.10, G06.11, G06.12, G06.13, G06.14, G06.15, G06.16, and G06.17). The hybrids were created as a result of crossing inbred lines of different origins, which were analyzed in detail by Sobiech et al. [31]. The origin of the lines used for crossbreeding is described below. Some of the lines analyzed were characterized by lines with flint grain of three different origins: F2 (a group related to the F2 line bred at INRA in France from the Lacaune population), EP1 (a group related to the EP1 line, bred in Spain from a population originating in the Pyrenees), and German Flint. The second part of the plant material consisted of dent-type kernels; these were derived from various groups of origin from the United States, specifically Iowa Stiff Stalk Synthetic (BSSS), Iowa Dent (ID), and Lancaster.

### 2.2. Methods

#### 2.2.1. Field Experiment

The experiment was established in plots of 10 m^2^, in three repetitions, in a randomized complete block design, at two localities (Smolice (51°42′58.904″ N, 17°13′29.13″ E) and Kobierzyce (50°58′19.411″ N, 16°55′47.323″ E)). Health evaluation was carried out on ten plants from each plot by calculating the percentage of stalks infected by Fusarium stalk rot.

#### 2.2.2. Weather Conditions

In 2022, the average rainfall in Smolice was 34.45 mm, which was 13.82 mm lower than the multi-year average. The highest rainfall was recorded in July (55 mm) and the lowest in March (15 mm). The average air temperature this year in Smolice was 9.54 °C, higher than the long-term average temperature by 0.8 °C. The warmest month in 2004 was August (20 °C), while the lowest temperature was recorded in December (1.1 °C). In 2022, the amount of rainfall and temperature were unfavorable during the initial development of maize plants. Despite the early sowing date, maize remained at the 2–3 leaf phase for a long time, during which purple discoloration was visible due to difficulties in taking up phosphorus from the soil. May had an abundance of rainfall, which had a positive effect on maize development. In Kobierzyce, the average rainfall in 2022 was 51.52 mm, 3.22 mm higher than the average multi-year rainfall. The highest rainfall was recorded in May, with 95.8 mm, and the lowest in March, with 21.6 mm. The average air temperature in Kobierzyce was 11.46 °C, which was higher than the multi-year average air temperature by 2.58 °C. The warmest month in 2022 in Kobierzyce, as in Smolice, was August (22 °C), and the coldest was December (1.5 °C).

#### 2.2.3. Determining the Degree of Fusarium Infection

Infection of maize plants by fungi *Fusarium verticillioides* was observed in natural conditions, in field experiments that were conducted in two localities in three repetitions. The plants were not artificially inoculated. In the stage of full maize maturity (BBCH 86), shortly before harvest, the number of plants with *Fusarium* wilt symptoms was determined, distinguishing the base of the stalk rot. The number of plants with symptoms of stalk rot was determined by lightly hitting the base of the stem with a foot, which caused fractures of the infected plants [32]. The degree of plant infection with stalk rot caused by fungi of the genus *Fusarium* spp. was determined during maize harvest based on visual inspection of the longitudinal section of the three lower internodes of the plant. The observation was made on ten plants. A 9-point scale was used [33], adopting the following parameters for individual degrees (even numbers mean indirect infection): 1—no visible symptoms (healthy plant), 3—one or two nodes turn brown and decay, 5—one or two internodes brownish, decaying, 7—more than two internodes brown and rotting, but pith tissue still exists around the vascular bundles, 9—all tissues decomposed, only conductive bundles visible. Next, for each repetition, based on observations of ten plants, the average percentage of stalk rot infected with *Fusarium* species was calculated.

#### 2.2.4. DNA Isolation

DNA isolation from 122 maize hybrids was carried out using a ready-made set of reagents from Symbios. The isolated DNA samples were subjected to next-generation sequencing. The concentration and purity of the isolated DNA samples were determined using a DS-11 spectrophotometer from DeNovix. The isolated DNA matrix was adjusted to a uniform concentration of 100 ng μL^−1^ by dilution with deionized distilled water.

#### 2.2.5. Genotyping

DArTseq technology, based on next-generation sequencing, was used for genotyping. Isolated DNA from the 122 maize hybrids tested at 50 µL at a concentration of 100 ng was transferred on two 96-well Eppendorf plates for analyses to identify SilicoDArT and SNP polymorphisms. The analyses were performed at Diversity Arrays Technology, University of Canberra, Australia. The methods used are described in detail on the Diversity Arrays Technology website: (https://www.diversityarrays.com/technology-and-resources/dartseq/, accessed on 15 January 2024).

#### 2.2.6. Association Mapping Using GWAS Analysis

Based on the results obtained from genotyping and phenotyping, association mapping was performed using GWAS analysis. Associations were determined for 122 analyzed maize hybrids. Genotypic data were obtained from DArTseq analysis, while phenotypic data were determined from observations of the percentage of stem infection and lodging of maize. Based on GWAS analysis, SilicoDArT and SNP markers with the highest level of significance, i.e., those that were most strongly associated with plant resistance to Fusarium stalk rot, were selected for further research. For the association analysis, only SilicoDArT and SNP sequences meeting the following criteria were selected: one SilicoDArT and/or SNP within a given sequence (69 nt), minor allele frequency (MAF) > 0.25, and the missing observation fractions <10%.

#### 2.2.7. Statistical Analysis and Association Mapping

Data were analyzed using analysis of variance in a model with fixed effects of location and random effects of genotypes and genotype × location interaction. Association mapping, based on SilicoDArT and SNP data and average trait values, was conducted separately for data from the two locations using the method based on the mixed linear model with a population structure estimated by eigenanalysis and modelled by random effects [34,35]. All analyses and visualizations of the results were performed in GenStat 23.1 [36], using procedure QSASSOCIATION. QSASSOCIATION performs a mixed model marker–trait association analysis (also known as linkage disequilibrium mapping) with data from a single-environment trial. To avoid false positives in association mapping studies, some form of control is necessary for the genetic relatedness. The model used was specified by the RELATIONSHIPMODEL=eigenanalysis option, which infers the underlying genetic substructure in the population by retaining the most significant principal components from the molecular marker matrix [37]; the scores of the significant axes are used as covariables in the mixed model, which effectively is an approximation to the structuring of the genetic variance covariance matrix by a coefficient of coancestry matrix (kinship matrix). Significance of association between Fusarium stalk rot and SilicoDArT and SNP markers was assessed using *p* values corrected for multiple testing using the Benjamini–Hochberg method.

#### 2.2.8. Physical Mapping

SilicoDArT and SNP marker sequences selected based on GWAS analysis were subjected to basic local alignment search tool (BLAST) analysis, which involves searching databases to find sequences with high homology to the selected silicoDArT and SNP marker sequences. Publicly available web browsers were used for this purpose, specifically CEPH Genotype database http://www.cephb.fr/en/cephdb/ (accessed on 15 January 2024), NCBI Map Viewer http://www.ncbi.nlm.nih.gov/projects/mapview/ (accessed on 15 January 2024), UCSC Genome Browser http://genome.ucsc.edu/ (accessed on 15 January 2024), and Ensembl Map View https://www.ensembl.org/index.html (accessed on 15 January 2024). The programs used were applied to indicate the chromosomal locations of the searched sequences, similar to the sequences analyzed, and to determine their physical location. The sequences of all genes located in the designated area on the chromosome were further analyzed.

#### 2.2.9. Functional Analysis of Gene Sequences

Functional analysis was carried out using Blast2GO 6.0 software. The sequences of all genes located in the chromosome regions determined by BLAST analysis were analyzed. The goal was to obtain information on the biological function of gene sequences located in the designated chromosome region.

#### 2.2.10. Design of Primers for Identified SilicoDArT and SNP Polymorphisms Related to Yield and Its Characteristics

The Primer 3 program was used to design the primers. The designed primers were used for a conventional PCR reaction; therefore, the PCR product size range from 50 to 500 bp was selected in the program. The melting temperature of the primers was selected as follows: min. 57.0 °C, opt. 60.0 °C, max. 63.0 °C, max. Tm difference 3. The other settings were not changed.

## 3. Results

### 3.1. Symptoms of Fusarium Stalk Rot

**Figure 1:**
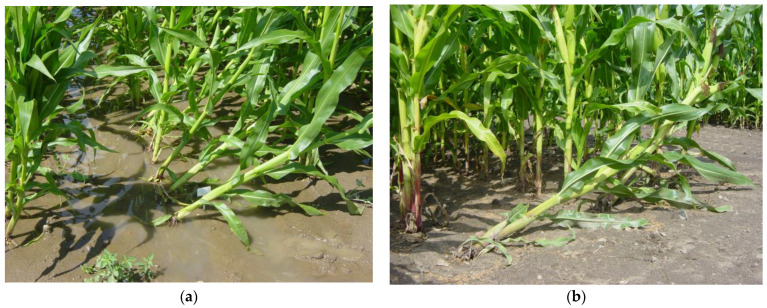
Symptoms of Fusarium stalk rot observed in: (**a**) Kobierzyce and (**b**) Smolice.

Typical symptoms of Fusarium stalk rot were observed on individual plants: rotting at roots, crown, and lower internodes; wilting of the plants, taking on a grayish-green hue, then turning tan; and light pink to tan discoloration, indicating splitting of the inner stalk (Figure 1). These symptoms were observed to varying degrees depending on the hybrid. Significantly higher Fusarium stalk rot infestation was observed in Kobierzyce than in Smolice.

### 3.2. Phenotyping

Detailed information on phenotypes such as mean, skewness, kurtosis, standard error, and coefficient of variation observed in each locality, as well as broad-sense heritability, is presented in Table 1. Significantly higher Fusarium stalk rot infestation was observed in Kobierzyce than in Smolice (Table 1). The large number of completely resistant plants (Fusarium stalk rot values equal to zero) in the experiment conducted in Smolice resulted in higher skewness, kurtosis, and coefficients of variation than in the experiment conducted in Kobierzyce. Broad-sense heritability for Fusarium stalk rot estimated from environments was 66.1 (Table 1).

**Figure 2:**
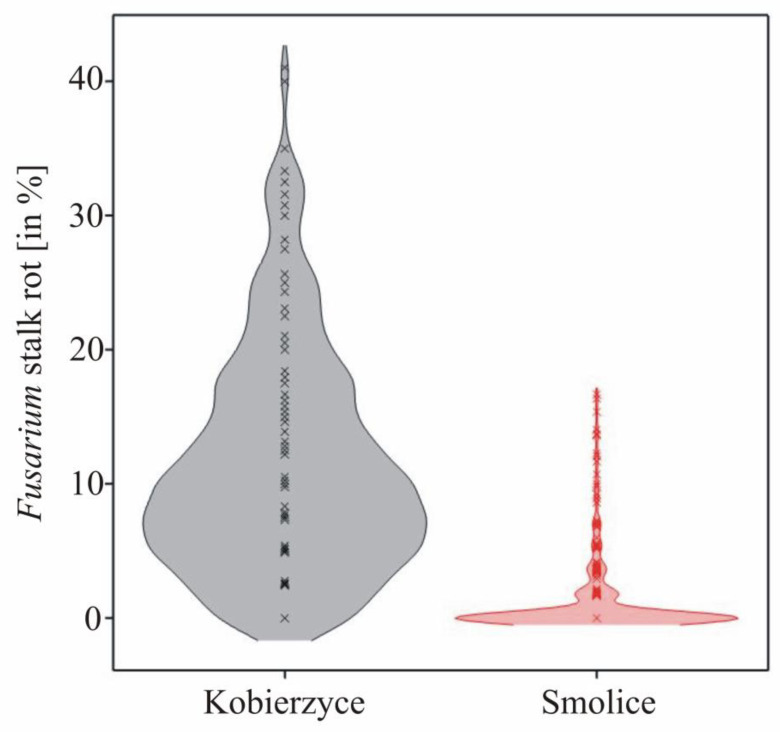
Density charts showing the distribution of the Fusarium stalk rot.

The observed Fusarium stalk rot was characterized by a normal distribution about both localities, which made it possible to carry out association mapping. Analysis of variance indicated that the main effects of genotype and location, as well as genotype × location interaction were significant for Fusarium stalk rot (Table 2). The differences between the locations were large (11.39% in Kobierzyce and 1.20% in Smolice), which was the result of large differences in weather conditions between the two locations (Figure 2).

The Fusarium stalk rot values ranged from 1.65% (for genotype G01.10) to 31.18% (for genotype G03.07) in Kobierzyce and from 0.00% (for 58 genotypes) to 6.36% (G05.03) in Smolice. On average for both localities, the Fusarium stalk rot values ranged from 0.82% (for genotype G01.10) to 17.08% (for genotype G03.07) (Table S1). The variability of the observed values (expressed in standard deviations) also differed between locations, from 0% to 16.65% in Kobierzyce and from 0% to 9.45% in Smolice (Table S1).

Despite very large differences between Fusarium stalk rot values, their correlation was observed between Kobierzyce and Smolice (*r* = 0.36, *p* < 0.001).

### 3.3. Genotyping

**Figure 3:**
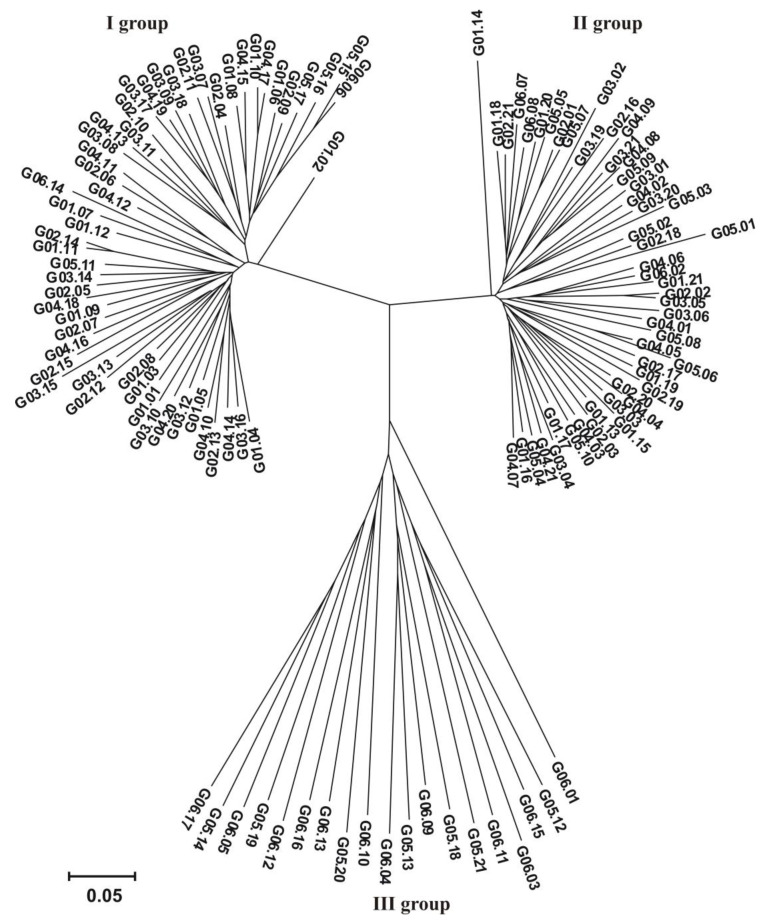
Dendrogram showing the genetic similarity between the 122 analyzed hybrids constructed based on the observation of 32,900 markers (26,234 SilicoDArT and 6666 SNP).

Illumina sequencing identified 60,436 SilicoDArT markers and 32,178 SNP markers (92,614 in total). For association mapping, 32,900 markers (26,234 SilicoDArT and 6666 SNP) meeting the criteria (MAF > 0.25 and the number of missing observations <10%) were used. Based on the identified SilicoDArT and SNP molecular markers, a dendrogram of genetic similarity between the 122 hybrids analyzed was prepared. The dendrogram distinguished three groups of similarity. Group I contained 53 hybrids, Group II contained 50 hybrids, and Group III contained 19 hybrids (Figure 3).

**Figure 4:**
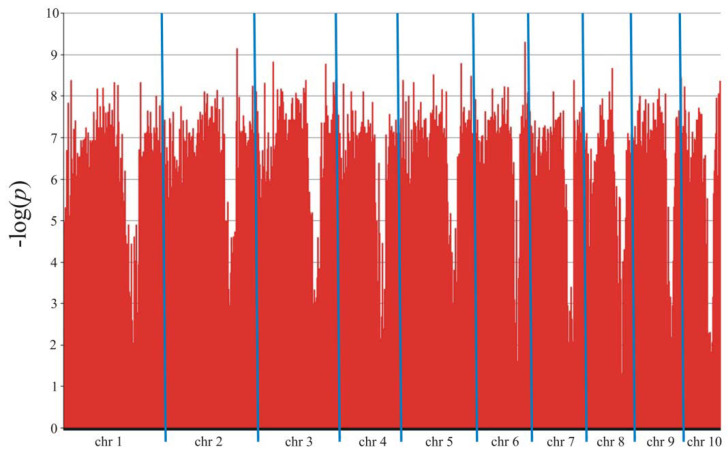
Manhattan plot for Fusarium stalk rot in Smolice.

**Figure 5:**
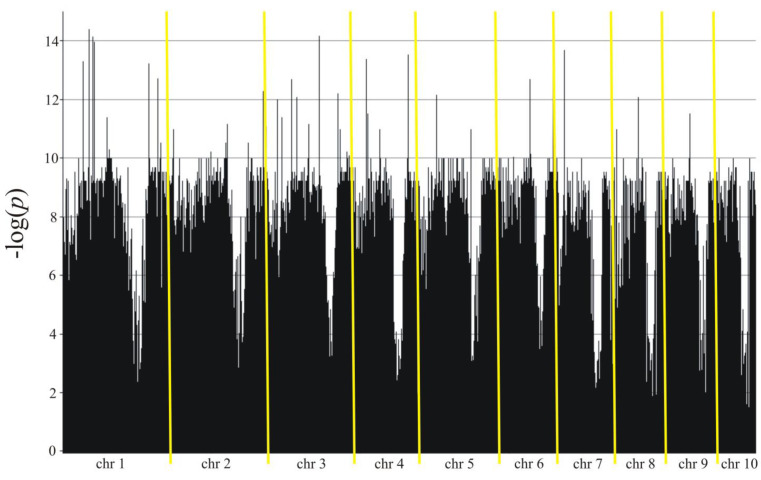
Manhattan plot for Fusarium stalk rot in Kobierzyce.

The association mapping identified 11,282 markers (8405 SilicoDArT and 2877 SNP) in Smolice (Table 3 and Figure 4) and 9439 markers (7148 SilicoDArT and 2291 SNP) in Kobierzyce (Table 3 and Figure 5) that were significant at a level of 0.05. The percentage of variation explained by each marker ranged from 2.4% to 34.4% in Smolice and from 2.4% to 27.0% in Kobierzyce (Table 3). A total of 8702 markers were statistically significant simultaneously in both localities. Of these, 91 were highly statistically significant (LOD > 7.5, Table 4). The percentage of variation explained by highly significant markers in both localities ranged from 22.0% (for four SilicoDArT markers: 9682713, 2432042, 2384252, and 4766257) to 30.5% (for SNP marker 4772836|F|0-17:C>A-17:C>A) in Smolice and from 22.0% (for four markers, two SilicoDArT (4766257 and 73750965) and two SNP (4586493|F|0-10:T>C-10:T>C and 25947704|F|0-38:C>T-38:C>T)) to 27.0% (for SNP marker 4771464|F|0-65:C>T-65:C>T) in Kobierzyce (Table 4).

Of the 8702 physically mapped statistically significant markers in both localities (Smolice and Kobierzyce), 10 were significantly associated with plant resistance to Fusarium stalk rot at both locations (Table 5). These were the markers with the highest statistical significance in both localities (LOD > 9.5 in Kobierzyce, LOD > 7.5 in Smolice) and the highest coefficients of determination (R^2^·100 > 27.4% in Kobierzyce, R^2^·100 > 22.1% in Smolice). Using the BLAST database, the location of the selected markers was determined and the associated candidate genes were provided. The next step was to design primers that would be used to identify the 10 selected markers. The sequences of the primers are shown in Table S2. The primers were designed to identify the analyzed markers using the conventional PCR method. Among the identified markers, two SNP markers located inside candidate genes play an important role. Marker 4772836 is located inside the serine/threonine-protein kinase bsk3 gene, while marker 4765764 is located inside the histidine kinase 1 gene. Both genes may be associated with plant resistance to Fusarium stalk rot.

## 4. Discussion

Maize is currently a leading crop in the global grain market, behind only rice and wheat [38]. Its largest producers are the United States and China, with 2018 production of 392 million tons and 257 million tons, respectively [39]. In terms of yield growth rate in production, maize dominates among cereal plants, with yield potential reaching 12–15 tons of dry matter per 1 ha. The high fertility of maize is the result of many favorable physiological and morphological traits of this species [40]. However, it should be remembered that yield levels are affected by weather conditions during a given growing season and local environmental conditions [41]. Maize has successfully acclimatized to Polish soil and climatic conditions [42,43]. The reason for this phenomenon is the introduction of linear hybrids into maize cultivation, providing access to varieties with an appropriate growing season. Maize, as a crop, is of great importance in the world, both in terms of utility and economy. This is due to the possibility of using virtually all of the plant’s biomass as feed, food, or industrial raw material [39].

Plants, including maize, are constantly exposed to many stress factors, both abiotic and biotic, that can affect their growth and development [41]. Biotic stress factors include viruses, bacteria, fungi, and insect pests. Often the consequence of stress factors is the occurrence of pathogenic diseases, which can interfere with normal plant growth, development, and function, and cause significant yield reduction [44]. Maize diseases occurring worldwide include common smut of maize, maize small leaf spot, maize sheath blight, common rust of maize, maize Helminthosporium, and maize stalk rot [45].

Fusarium stalk rot of maize is caused by fungi of the genus *Fusarium*. Damage caused by European maize stalk rot promotes the development of the pathogenic fungus [46]. Characteristic traits of the disease are wilting and drying of leaf blades, weakening of the stem, and often lodging [47]. In addition, *Fusarium* fungi increase the content of mycotoxins, which in turn disqualify grain from further processing [48,49,50].

In our own research, the field experiment made it possible to perform and analyze measurements of the degree of *Fusarium* infection of maize stalks. The observed trait was characterized by a normal distribution at both localities, which made it possible to perform association mapping. Analysis of variance indicated that the main effects of genotype and location, as well as genotype and location interaction, were significant for Fusarium stalk rot.

The most important mycotoxins include aflatoxins, fumonisins, ochratoxin A, deoxynivalenol, zearalenone, and ergot alkaloids, which are mainly produced by the genera *Aspergillus*, *Penicillium*, *Fusarium*, and *Claviceps* [6]. Selection of resistant genotypes based on their phenotype can be unreliable and is time- and labor-consuming. Phenotyping maize for *Fusarium* resistance requires field trials conducted in several environments due to the high interaction of genotype and environment [51]. In addition, proper management of inoculation timing is required due to the diversity of genotypes in terms of flowering [52]. Currently, the most effective and fastest methods to support selection are transcriptomic and genomic association studies (GWAS). These are useful tools for identifying candidate genes, especially when combined with QTL mapping to map and validate loci for quantitative traits [53]. Combining these methods has allowed us to overcome their individual limitations [54,55].

Our study identified 60,436 SilicoDArT markers and 32,178 SNP markers (92,614 in total). For association mapping, 32,900 markers (26,234 silicoDArT and 6666 SNP) were selected, meeting assumptions about the distribution of values (MAF > 0.25) and the number of missing observations (<10%). The differentiation of hybrids at the genetic level makes the association mapping results obtained even more reliable and will be solid selection tools in maize breeding. The association mapping identified 11,282 markers (8405 silicoDArT and 2877 SNP) in the Smolice experiment and 9439 markers (7148 silicoDArT and 2291 SNP) in the Kobierzyce experiment. Of the 8702 markers associated with physical mapping, 10 were selected that were statistically significant in both localities (Smolice and Kobierzyce). The identification of ten markers coupled to specific genes with specific functions provides information about the genetic basis, as well as pointing to the molecular mechanisms controlling resistance to Fusarium stalk rot. The use of maize hybrids as an association population makes it possible to create favorable conditions for increasing maize resistance to *F. verticillioides* through genomics-assisted breeding [56,57].

In recent years, many authors have attempted to identify molecular markers associated with functionally important traits in maize. Using next-generation sequencing, Sobiech et al. [58] identified markers associated with *Fusarium* resistance in maize plants. Bocianowski et al. [59] used NGS technology and association mapping to identify markers associated with the heterosis effect in maize. A similar study was conducted by Tomkowiak et al. [60], who identified six SNP markers (1818, 14506, 2317, 3233, 11657, and 12812) located within genes, on chromosomes 8, 9, 7, 3, 5, and 1, respectively, associated with maize yield. Other authors [61] identified four genes (the isoform × 2 gene of sucrose synthase 4, the isoform × 1 gene of phosphoinositide phosphatase, the putative isoform × 1 gene of the SET domain-containing protein family, and the grx_c8–glutaredoxin subgroup iii gene) which significantly regulate the level of vigor and germination of maize seeds.

Stalk rot is a destructive disease in maize and occurs worldwide. The genetic mechanism of maize stalk rot is complex. It is caused by one or more fungal pathogen(s). The causative agents, including *F. verticillioides*, *Fusarium moniliforme*, and *Fusarium proliferatum*, are soil-borne fungus, meaning that it is difficult to control stalk rot. Thus, it is necessary to understand the genetic basis of stalk rot and to breed resistant varieties. The stalk rot resistance in maize is controlled by polygene and the additive genetic effects among the genes [62,63]. Until now, a number of quantitative trait loci (QTLs) associated with maize stalk rot have been identified [64,65,66]. In the study presented here, of the ten markers selected, two of them are located inside genes. SNP marker 4772836, located on chromosome 2, is located inside the serine/threonine-protein kinase bsk3 gene, while SNP marker 4765764, located on chromosome 5, is inside the histidine kinase 1 gene. Histidine kinases are typically trans-membrane proteins of the transferase class that play a role in signal transduction across the cellular membrane. The vast majority of histidine kinases (HKs) are homodimers that exhibit autokinase, phosphotransfer, and phosphatase activity [67]. Unlike other classes of protein kinases, HKs are usually parts of a two-component signal transduction mechanism, in which the HK transfers a phosphate group from ATP to a histidine residue inside the kinase, then to an aspartate residue in the receiver domain of the response regulator protein (or sometimes on the kinase itself). Histidine kinases have been implicated in cytokinin and ethylene signaling, in regulation responses to environmental stress [68]. It can be speculated that they are also involved in the immune response associated with *Fusarium* infection of plants.

The second gene analyzed, a probable serine/threonine kinase, acts as a positive regulator of brassinosteroid (BR) signaling. Brassinosteroids, another class of growth-promoting regulator, regulate many aspects of plant growth and development, including cell expansion and elongation (in combination with auxin) and vascular differentiation [69,70,71,72,73]. The necessity of brassinosteroids in plant growth and development has been demonstrated by the identification of many BR-deficient dwarf mutants and subsequent BRs treating experiments [74]. Brassinosteroid-signaling kinases (BSKs) are critical in BRs signal transduction. BSK3 is the only BSK member involved in BR-mediated plant root growth [75].

To confirm the involvement of the two candidate genes in the immune response to infection of maize plants by *Fusarium* fungi, their expression will be analyzed. If the involvement of these genes in the immune reaction is confirmed, SNP markers associated with them can be used to select resistant varieties.

## 5. Conclusions

In maize cultivation, it is crucial to reduce the severity of diseases that cause a drastic decrease in yields. Excessive use of chemical protection negatively affects the natural environment, so the best method of crop protection is to use varieties with genetically determined resistance in breeding. Understanding the genetic basis and molecular mechanisms controlling *Fusarium* resistance is a key requirement for developing maize varieties with enhanced resistance. Because resistance to *Fusarium* fungi is a quantitative trait based on a distributed architecture of multiple genes with small effectors, the best approach for future molecular breeding is MAS. Using next-generation sequencing (NGS), two significant markers were identified in candidate genes associated with *Fusarium* resistance in maize plants. SNP marker 4772836, located on chromosome 2, is inside the serine/threonine-protein kinase bsk3 gene, while SNP marker 4765764, located on chromosome 5, is inside the histidine kinase 1 gene. If the involvement of these genes in the immune response is confirmed, SNP markers associated with them can be used to select resistant varieties.

## Figures and Tables

**Table 1:** Statistical summary of phenotypes of 122 maize hybrids for Fusarium stalk rot.

Location	Repeat	Mean	Skewness	Kurtosis	Coefficient of Variation (%)	Broad-Sense Heritability
Kobierzyce	Repeat 1	11.18	−0.81	0.22	23.12	66.1
	Repeat 2	11.09	0.17	−0.55	29.94	
	Repeat 3	11.90	−0.37	0.60	28.79	
	Mean	11.39	−0.50	0.47	21.17	
Smolice	Repeat 1	1.25	−1.49	0.91	50.02	
	Repeat 2	1.06	−1.15	0.88	40.47	
	Repeat 3	1.34	−1.27	−0.95	55.20	
	Mean	1.22	−1.12	0.59	28.47	

**Table 2:** Results of two-way analysis of variance for Fusarium stalk rot.

Source of Variation	The Number of Degrees of Freedom	Sum of Square	Mean Square	*F* Statistics
Genotype	121	8468.68	69.99	2.45 ***
Location	1	18,988.97	18,988.97	664.68 ***
Genotype × Location	121	5765.31	47.65	1.67 ***
Residual	488	13,941.44	28.57	
Total	731	47,164.40		

*** *p* < 0.001.

**Table 3:** SilicoDArT and SNP molecular markers significantly associated with maize plant resistance to Fusarium stalk rot in Smolice and Kobierzyce (significant associations selected at *p* < 0.001 with correction for Benjamini–Hochberg multiple testing).

Type of Markers		All	SilicoDArT	SNP
Smolice				
The number of markers		11,282	8405	2877
Negative	Numbers	5800	4336	1464
	Effects	−17.06–−2.14	−13.88–−2.14	−17.06–−2.14
	Percentage variance accounted for	2.4–31.4	2.4–31.4	2.4–30.5
	LOD	1.30–12.70	1.30–16.70	1.30–11.10
Positive	Numbers	5482	4069	1413
	Effects	2.14–8.55	2.14–8.55	2.17–7.37
	Percentage variance accounted for	2.4–34.4	2.4–33.7	2.4–34.4
	LOD	1.30–14.40	1.30–14.40	1.30–13.53
Kobierzyce				
The number of markers		9439	7148	2291
Negative	Numbers	4523	3437	1086
	Effects	−6.49–−0.78	−6.79–−0.777	−4.38–−0.784
	Percentage variance accounted for	2.4–27.0	2.4–25.7	2.4–27.0
	LOD	1.30–9.30	1.30–8.82	1.30–9.30
Positive	Numbers	4916	3711	1205
	Effects	0.779–2.214	0.779–2.214	0.788–2.173
	Percentage variance accounted for	2.4–25.3	2.4–25.3	2.4–23.9
	LOD	1.30–8.68	1.30–8.68	1.31–8.17

**Table 4:** Ninety-one markers highly statistically significant (LOD > 7.5) simultaneously in both localities: Kobierzyce and Smolice.

Chromosome	Marker Type	CloneID	Kobierzyce			Smolice		
			Estimate	Percen ^1^	LOD	Estimate	Percen	LOD
1	SilicoDArT	29628241	5.902	23.4	8.02	2.167	24.3	8.33
1	SilicoDArT	82348823	6.031	24.4	8.34	2.167	24.2	8.27
1	SilicoDArT	24026805	6.219	26.0	8.92	2.156	24.0	8.20
1	SNP	4772298|F|0-68:A>G-68:A>G	−6.032	24.5	8.40	−2.127	23.4	8.00
1	SilicoDArT	2488934	−5.894	22.1	7.54	−2.166	22.9	7.84
1	SNP	2439850|F|0-12:A>C-12:A>C	−6.061	24.9	8.52	−2.092	22.7	7.76
1	SilicoDArT	4774875	5.823	22.9	7.83	2.092	22.7	7.76
1	SilicoDArT	9626410	6.623	29.9	10.30	2.073	22.3	7.62
1	SNP	2529315|F|0-56:T>G-56:T>G	−6.163	25.8	8.82	−2.073	22.3	7.62
1	SilicoDArT	7054095	−6.361	27.5	9.52	−2.073	22.3	7.61
1	SilicoDArT	7051768	6.122	25.4	8.70	2.071	22.2	7.60
2	SNP	4772836|F|0-17:C>A-17:C>A	−6.722	30.5	11.10	−2.148	23.7	8.11
2	SNP	4582743|F|0-42:G>A-42:G>A	−6.073	24.6	8.42	−2.135	23.3	7.97
2	SilicoDArT	9694283	5.932	23.8	8.12	2.100	22.8	7.81
2	SilicoDArT	82349036	6.234	26.4	9.05	2.092	22.7	7.76
2	SilicoDArT	2395963	5.823	22.9	7.83	2.092	22.7	7.76
2	SilicoDArT	2382023	5.823	22.9	7.83	2.092	22.7	7.76
2	SilicoDArT	9703016	5.823	22.9	7.83	2.092	22.7	7.76
2	SilicoDArT	25942787	5.764	22.3	7.63	2.088	22.5	7.69
2	SilicoDArT	9633940	6.000	24.1	8.24	2.093	22.5	7.68
2	SilicoDArT	9718212	5.965	24.1	8.24	2.073	22.3	7.61
2	SilicoDArT	4778784	−6.347	27.4	9.40	−2.072	22.3	7.61
2	SilicoDArT	29619311	5.742	22.2	7.59	2.066	22.1	7.54
3	SNP	4772456|F|0-52:T>G-52:T>G	−5.802	22.5	7.68	−2.227	25.6	8.77
3	SNP	4585365|F|0-34:T>C-34:T>C	−5.979	23.8	8.15	−2.186	24.5	8.39
3	SilicoDArT	9682713	−5.718	22.0	7.52	−2.138	23.7	8.11
3	SilicoDArT	9717799	5.981	24.0	8.22	2.140	23.6	8.08
3	SilicoDArT	77157803	5.877	23.2	7.92	2.140	23.6	8.08
3	SNP	2433795|F|0-30:G>C-30:G>C	5.996	24.2	8.29	2.129	23.4	8.02
3	SilicoDArT	77158337	5.768	22.5	7.68	2.111	23.1	7.91
3	SilicoDArT	29621917	−5.998	24.4	8.36	−2.101	23.0	7.85
3	SilicoDArT	5583810	6.071	24.9	8.54	2.100	22.8	7.81
3	SNP	4774080|F|0-65:C>T-65:C>T	−5.868	23.3	7.95	−2.092	22.7	7.76
3	SNP	4774088|F|0-45:G>A-45:G>A	−5.868	23.3	7.95	−2.092	22.7	7.76
3	SNP	7048352|F|0-24:A>G-24:A>G	−5.737	22.2	7.59	−2.092	22.7	7.76
3	SNP	5586725|F|0-40:C>A-40:C>A	−5.761	22.2	7.60	−2.099	22.7	7.75
3	SilicoDArT	82349016	6.017	24.5	8.38	2.074	22.3	7.60
3	SilicoDArT	4768318	−5.845	23.1	7.90	−2.067	22.2	7.58
3	SilicoDArT	34685358	6.096	25.2	8.64	2.066	22.2	7.57
3	SNP	2403483|F|0-62:C>A-62:C>A	−5.881	23.4	8.00	−2.066	22.2	7.57
3	SNP	4771426|F|0-54:A>G-54:A>G	6.129	25.3	8.66	2.072	22.1	7.55
3	SNP	4592970|F|0-41:T>C-41:T>C	−5.924	23.6	8.08	−2.067	22.1	7.54
3	SNP	4586493|F|0-10:T>C-10:T>C	−5.734	22.1	7.56	−2.063	22.0	7.52
4	SilicoDArT	70092308	5.823	22.9	7.83	2.092	22.7	7.76
4	SilicoDArT	9680684	5.823	22.9	7.83	2.092	22.7	7.76
4	SilicoDArT	25001071	5.854	23.1	7.90	2.078	22.4	7.64
4	SilicoDArT	25942566	5.747	22.2	7.60	2.078	22.4	7.64
4	SilicoDArT	25004669	−6.261	26.3	9.05	−2.078	22.1	7.56
5	SNP	4583014|F|0-63:A>G-63:A>G	−5.998	24.0	8.20	−2.235	25.6	8.80
5	SNP	2536415|F|0-25:C>T-25:C>T	−5.926	23.6	8.09	−2.187	24.8	8.48
5	SilicoDArT	4578971	−5.881	23.2	7.93	−2.177	24.5	8.38
5	SilicoDArT	2401113	−6.205	26.0	8.92	−2.168	24.3	8.33
5	SilicoDArT	29628894	6.294	26.8	9.22	2.148	23.9	8.17
5	SilicoDArT	2432042	−5.722	22.0	7.51	−2.128	23.4	8.01
5	SilicoDArT	2499631	−6.401	27.7	9.52	−2.108	23.0	7.85
5	SilicoDArT	9690308	6.326	27.2	9.40	2.092	22.7	7.76
5	SilicoDArT	9681187	6.145	25.6	8.77	2.092	22.7	7.76
5	SilicoDArT	2539842	6.016	24.5	8.38	2.081	22.4	7.66
5	SilicoDArT	4776114	6.062	24.8	8.51	2.081	22.4	7.66
5	SilicoDArT	4779143	6.465	28.5	9.70	2.066	22.2	7.57
5	SilicoDArT	2532640	6.355	27.5	9.52	2.066	22.2	7.57
6	SNP	4771464|F|0-65:C>T-65:C>T	−6.251	25.7	8.82	−2.309	27.0	9.30
6	SilicoDArT	5587687	5.989	24.1	8.24	2.158	24.0	8.22
6	SilicoDArT	4770044	−6.073	24.6	8.42	−2.160	23.9	8.17
6	SilicoDArT	5588627	5.855	23.1	7.90	2.119	23.3	7.96
6	SNP	4770865|F|0-46:C>T-46:C>T	−6.028	24.6	8.41	−2.101	22.9	7.81
6	SilicoDArT	2530960	5.794	22.6	7.73	2.098	22.8	7.79
6	SilicoDArT	9698143	6.459	28.2	9.70	2.084	22.4	7.67
6	SNP	4764810|F|0-20:T>C-20:T>C	−6.424	27.2	9.40	−2.107	22.3	7.63
6	SilicoDArT	4775726	6.185	26.0	8.92	2.073	22.3	7.63
6	SilicoDArT	24029725	5.850	23.1	7.90	2.073	22.3	7.61
6	SNP	25947704|F|0-38:C>T-38:C>T	−6.129	25.4	8.72	−2.061	22.0	7.51
7	SNP	67225764|F|0-56:C>G-56:C>G	−6.228	26.3	9.05	−2.073	22.3	7.61
7	SNP	2523212|F|0-36:C>T-36:C>T	−5.778	22.5	7.70	−2.072	22.3	7.61
8	SilicoDArT	9694332	5.899	23.5	8.03	2.081	22.4	7.66
8	SilicoDArT	9698173	5.991	24.3	8.33	2.066	22.2	7.57
8	SilicoDArT	4766257	5.708	22.0	7.52	2.060	22.0	7.52
9	SNP	2475427|F|0-37:A>G-37:A>G	−6.020	24.2	8.27	−2.195	24.7	8.46
9	SilicoDArT	77157588	5.888	22.8	7.81	2.156	23.6	8.06
9	SilicoDArT	4584767	−5.958	23.6	8.09	−2.140	23.4	8.01
9	SilicoDArT	7049648	−6.051	24.7	8.46	−2.083	22.4	7.66
9	SilicoDArT	2384252	5.712	22.0	7.52	2.072	22.3	7.61
9	SilicoDArT	25947915	5.811	22.5	7.71	2.082	22.2	7.60
9	SilicoDArT	73750965	5.912	23.5	8.05	2.065	22.0	7.52
10	SNP	9667431|F|0-38:G>C-38:G>C	6.403	27.8	9.52	2.120	23.3	7.96
10	SilicoDArT	2575390	5.817	22.9	7.82	2.085	22.6	7.72
10	SilicoDArT	9669953	−5.857	22.8	7.80	−2.095	22.4	7.66
10	SilicoDArT	5584917	6.512	28.9	10.00	2.073	22.3	7.62
10	SilicoDArT	2408858	−5.816	22.6	7.72	−2.084	22.3	7.61
10	SilicoDArT	7054499	6.179	25.9	8.89	2.066	22.2	7.57
10	SilicoDArT	25002574	6.455	28.2	9.70	2.070	22.1	7.56

^1^ Percentage variance accounted for.

**Table 5:** Characteristics and location of markers significantly related to plant resistance to Fusarium stalk rot.

Marker	Marker Type	Chromosome	Marker Location	Candidate Genes
4772836	SNP	Chr 2	1002990	serine/threonine-protein kinase bsk3
9626410	SilicoDArT	Chr 1	147389719	45,560 bp at 5′ side: uncharacterized protein loc100276421 isoform ×1 74,882 bp at 3′ side: ubiquitin carboxyl-terminal hydrolase 15 isoform ×1
5584917	SilicoDArT	Chr 10	137279593	uncharacterized protein loc103641914 isoform ×1 uncharacterized protein loc103641914 isoform ×2
77157434	SilicoDArT	Chr 2	210848643	14,188 bp at 5′ side: formin-like protein 11 23,892 bp at 3′ side: uncharacterized protein loc100273879
9698143	SilicoDArT	Chr 6	168628814	440 bp at 5′ side: uncharacterized protein loc100273604 86,823 bp at 3′ side: uncharacterized protein loc100382826 precursor
2499631	SilicoDArT	Chr 5	223354035	33,662 bp at 5′ side: putative protein of unknown function (duf640) domain fami 10,443 bp at 3′ side: nac domain-containing protein 92
21699135	SilicoDArT	Chr 5	148291902	3967 bp at 5′ side: uncharacterized protein loc103627005 11,771 bp at 3′ side: uncharacterized protein loc100279673 precursor
7054095	SilicoDArT	Chr 1	192290825	1016 bp at 5′ side: abc transporter c family member 10 24,169 bp at 3′ side: probable lipoxygenase 8, chloroplastic
4779143	SilicoDArT	Chr 5	32830342	20,659 bp at 5′ side: putative disease resistance protein rga4 517 bp at 3′ side: uncharacterized protein loc101027116 precursor
4765764	SNP	Chr 5	213770951	histidine kinase 1

## Data Availability

The data presented in this study are available on request from the corresponding author. The data are not publicly available due to legal reason.

## Supplementary Materials

The following supporting information can be downloaded at: https://www.mdpi.com/article/10.3390/genes15010106/s1, Table S1: Mean values and standard deviation (s.d.) of Fusarium stalk rot for particular genotypes in two locations, as well as averages of the locations; Table S2: Sequences of designed primers used to identify newly selected markers significantly associated with plant Fusarium stalk rot resistance.
