# Supplementary material for: Using NGS Technology and Association Mapping to Identify Candidate Genes Associated with Fusarium Stalk Rot Resistance
Source: Genes (Basel). 2024 Jan 16;15(1):106. doi: 10.3390/genes15010106 (PMC10815114; doi:10.3390/genes15010106)
Supplement: Supplementary file 1 [file genes-15-00106-s001.zip › genes-2812598-supplementary.pdf]

Article

Using NGS Technology and Association Mapping to Identify Candidate Genes Associated with Fusarium Stalk Rot Resistance

Jan Bocianowski [1,*]

[1] Department of Mathematical and Statistical Methods, Poznań University of Life Sciences, Wojska Polskiego 28, 60-637 Poznań, Poland; jan.bocianowski@up.poznan.pl

* Correspondence: jan.bocianowski@up.poznan.pl; Tel.: +48 61 8487143

Supplementary Materials:

Table S1. Mean values and standard deviation (s.d.) of Fusarium stalk rot for particular genotypes in two locations as well as for the average of the locations.

Location	Kobierzyce		Smolice		Average		Location	Kobierzyce		Smolice		Average	
Genotype	Mean	s.d.	Mean	s.d.	Mean	s.d.	Genotype	Mean	s.d.	Mean	s.d.	Mean	s.d.
G01.01	5.83	2.89	0.00	0.00	2.92	3.68	G03.20	5.83	8.04	0.61	1.05	3.22	5.87
G01.02	6.84	3.92	0.00	0.00	3.42	4.49	G03.21	25.98	7.39	5.45	9.45	15.72	13.56
G01.03	6.80	1.57	1.82	3.15	4.31	3.52	G04.01	12.68	2.24	0.00	0.00	6.34	7.09
G01.04	9.11	10.12	0.00	0.00	4.55	8.11	G04.02	11.67	1.44	5.85	1.92	8.76	3.53
G01.05	7.50	2.50	0.57	0.99	4.04	4.16	G04.03	8.33	8.04	0.62	1.07	4.48	6.65
G01.06	5.83	1.44	0.00	0.00	2.92	3.32	G04.04	4.19	2.87	1.16	1.00	2.67	2.54
G01.07	15.11	8.97	1.25	1.09	8.18	9.50	G04.05	1.67	2.89	0.61	1.05	1.14	2.03
G01.08	4.17	1.45	0.00	0.00	2.09	2.46	G04.06	11.86	6.43	2.44	2.13	7.15	6.71
G01.09	10.22	6.75	0.00	0.00	5.11	7.04	G04.07	3.33	3.82	0.00	0.00	1.67	3.03
G01.10	1.65	1.43	0.00	0.00	0.82	1.28	G04.08	14.46	7.48	5.18	7.46	9.82	8.39
G01.11	14.53	7.84	0.58	1.01	7.56	9.13	G04.09	5.00	4.33	0.00	0.00	2.50	3.87
G01.12	8.55	4.18	0.00	0.00	4.28	5.38	G04.10	12.52	11.43	3.95	4.36	8. 23	9.05
G01.13	7.61	8.56	0.00	0.00	3.81	6.84	G04.11	7.65	8.92	0.58	1.01	4.12	6.87
G01.14	4.27	3.92	0.00	0.00	2.14	3.41	G04.12	5.09	5.00	0.00	0.00	2.54	4.22
G01.15	23.72	2.80	0.00	0.00	11.86	13.11	G04.13	3.33	2.89	0.00	0.00	1.67	2.58
G01.16	11.97	7.83	0.00	0.00	5.98	8.22	G04.14	16.05	7.59	0.57	0.99	8.31	9.76
G01.17	15.83	3.82	3.33	5.77	9.58	8.13	G04.15	13.33	16.65	0.00	0.00	6.67	12.81
G01.18	8.38	6.23	0.72	1.25	4.55	5.81	G04.16	23.61	9.22	1.85	3.21	12.73	13.42
G01.19	16.89	7.03	0.00	0.00	8.44	10.26	G04.17	10.83	8.04	0.00	0.00	5.42	7.81
G01.20	16.09	12.78	0.00	0.00	8.05	11.96	G04.18	19.38	8.27	3.57	6.18	11.48	10.85
G01.21	16.77	9.40	0.00	0.00	8.39	10.94	G04.19	10.00	2.50	0.71	1.23	5.36	5.39
G02.01	1.67	1.44	0.00	0.00	0.83	1.29	G04.20	21.03	3.07	1.23	2.14	11.13	11.10
G02.02	10.00	2.50	3.61	3.64	6.81	4.48	G04.21	9.40	10.36	1.36	2.36	5.38	8.04
G02.03	2.50	0.00	0.00	0.00	1.25	1.37	G05.01	5.83	1.44	1.96	2.10	3.90	2.66
G02.04	15.06	8.94	0.00	0.00	7.53	10.00	G05.02	11.84	5.49	0.00	0.00	5.92	7.36
G02.05	13.42	7.50	0.00	0.00	6.71	8.75	G05.03	15.11	4.24	6.36	6.06	10.74	6.70
G02.06	15.83	1.44	0.58	1.01	8.21	8.43	G05.04	9.07	3.69	0.00	0.00	4.53	5.49

G02.07	11.10	6.84	0.00	0.00	5.55	7.46	G05.05	15.17	5.26	4.85	4.32	10.01	7.10
G02.08	21.90	5.60	5.64	5.84	13.77	10.27	G05.06	4.23	3.90	1.26	2.18	2.74	3.26
G02.09	4.25	3.85	0.00	0.00	2.13	3.37	G05.07	9.65	8.46	2.30	3.98	5.98	7.15
G02.10	10.96	7.33	4.68	8.11	7.82	7.72	G05.08	6.67	2.89	1.75	3.04	4.21	3.78
G02.11	7.91	7.31	0.62	1.07	4.26	6.15	G05.09	15.83	10.10	3.07	2.76	9.45	9.63
G02.12	16.71	13.70	1.89	1.96	9.30	11.94	G05.10	5.88	3.81	0.62	1.07	3.25	3.81
G02.13	8.33	2.89	0.00	0.00	4.17	4.92	G05.11	16.67	11.82	2.90	3.50	9.78	10.84
G02.14	17.27	6.23	1.19	2.06	9.23	9.74	G05.12	13.14	6.84	0.57	0.99	6.86	8.16
G02.15	4.30	5.33	0.00	0.00	2.15	4.11	G05.13	21.67	7.22	0.67	1.16	11.17	12.40
G02.16	8.51	3.05	0.00	0.00	4.26	5.05	G05.14	14.17	9.47	0.00	0.00	7.08	9.80
G02.17	6.67	3.82	0.00	0.00	3.33	4.38	G05.15	21.94	10.55	1.19	2.06	11.57	13.25
G02.18	11.84	2.75	0.58	1.01	6.21	6.44	G05.16	3.42	5.92	0.00	0.00	1.71	4.19
G02.19	5.04	0.08	0.00	0.00	2.52	2.76	G05.17	11.56	5.38	0.00	0.00	5.78	7.19
G02.20	17.68	12.84	1.72	2.99	9.70	12.08	G05.18	6.10	4.08	0.00	0.00	3.05	4.22
G02.21	3.42	2.97	0.00	0.00	1.71	2.65	G05.19	12.65	5.23	4.97	2.72	8.81	5.62
G03.01	5.00	4.33	3.02	2.20	4.01	3.26	G05.20	17.50	9.01	2.21	1.92	9.85	10.21
G03.02	10.94	8.37	0.63	1.09	5.79	7.77	G05.21	10.92	1.37	2.35	2.67	6.64	5.07
G03.03	11.82	10.24	0.00	0.00	5.91	9.15	G06.01	9.34	10.38	1.82	3.15	5.58	8.00
G03.04	1.67	2.89	1.17	2.03	1.42	2.25	G06.02	6.84	5.93	0.00	0.00	3.42	5.30
G03.05	19.57	10.48	6.28	8.07	12.93	11.09	G06.03	9.43	5.65	1.73	1.73	5.58	5.63
G03.06	19.62	13.23	0.00	0.00	9.81	13.62	G06.04	20.21	13.80	0.00	0.00	10.11	14.10
G03.07	31.18	16.18	2.98	5.16	17.08	18.81	G06.05	7.54	6.59	0.00	0.00	3.77	5.87
G03.08	24.32	7.44	0.00	0.00	12.16	14.13	G06.06	17.50	5.00	1.13	0.98	9.32	9.53
G03.09	14.92	8.73	0.00	0.00	7.46	9.86	G06.07	9.38	6.49	0.57	0.99	4.98	6.36
G03.10	4.23	3.90	0.57	0.99	2.40	3.24	G06.08	10.00	2.50	1.15	1.00	5.58	5.14
G03.11	13.33	8.04	0.00	0.00	6.67	8.90	G06.09	9.19	7.09	0.00	0.00	4.59	6.74
G03.12	5.92	5.31	1.74	1.76	3.83	4.21	G06.10	14.98	2.36	1.80	1.85	8.39	7.47
G03.13	15.61	14.03	4.55	7.88	10.08	11.84	G06.11	14.17	11.55	0.00	0.00	7.08	10.66
G03.14	15.46	4.85	0.00	0.00	7.73	9.00	G06.12	25.15	7.28	0.60	1.03	12.87	14.23
G03.15	15.00	5.00	5.32	6.03	10.16	7.26	G06.13	14.49	7.79	0.00	0.00	7.25	9.34
G03.16	5.88	1.41	0.00	0.00	2.94	3.34	G06.14	11.84	8.17	0.00	0.00	5.92	8.29
G03.17	13.55	1.30	6.16	9.14	9.86	7.11	G06.15	10.92	6.28	0.00	0.00	5.46	7.18
G03.18	5.04	2.50	0.00	0.00	2.52	3.18	G06.16	5.77	1.34	0.00	0.00	2.89	3.27
G03.19	15.28	9.29	3.64	3.57	9.46	8.96	G06.17	5.83	2.89	0.00	0.00	2.92	3.68
Location	11.39	8.33	1.20	2.79									
$LSD_{0.05}$ – Genotype: 6.06; Location: 0.78; Genotype × Location: 8.58													

LSD – Least Significant Difference.

Table S2. Sequences of designed primers used to identify newly selected markers significantly associated with plant Fusarium stalk rot resistance.

Marker	Primer sequence		Melting temper-ature (°C)	Product size (bp)
	Forward	Reverse		
4772836	GGTGGTTTTACCCCCTGCAG	TATTTGCAGGCCCTTGACCT	59	281
9626410	AGCAATTTCTCCAGAGTCTGATG	CATGCATTTTTCTGCATTGGGC	61	50
5584917	TATTGAAGAGAGATATGA-TAATCGCTGCAG	GTTCAAATAACTCGCAAAA-GACTCG	58	78
77157434	CGGACCGTATTACCCGGTTA	AATTTCCGCGGTACCGAGGC	55	270
9698143	ACCGTGGCTAATCCGGTTAT	GGCATTCCGGGTAATCCGTT	59	350
2499631	CGGTTCCAATTGGGATTACC	CCTGGACCGGCTTTACAATC	61	145
21699135	CCGATACTGCATGCTCTGCG	CCTCTGTTTGGCGTAGGTGA	59	64
7054095	GTCGACGACGAACCCTGCAG	CCAATATCCGGCGGACAGAC	61	55
4779143	AATACCCTGGGTCCGGTAA	TTACCGGGTCCAACCTGGC	58	180
4765764	TTTTTTCCTTCTTGCTGCAG	CCTCGTTCTGTGAACTGGAA	61	263
